# A niche construction approach on the central Netherlands covering the last 220,000 years

**DOI:** 10.1007/s12685-015-0141-y

**Published:** 2015-08-29

**Authors:** Don F. A. M. van den Biggelaar, Sjoerd J. Kluiving

**Affiliations:** Institute for Geo- and Bioarchaeology, Faculty of Earth and Life Sciences, VU University Amsterdam, De Boelelaan 1085, 1081 HV Amsterdam, The Netherlands; Department of Archaeology, Ancient History of Mediterranean Studies and Near Eastern Studies, Faculty of Arts, VU University Amsterdam, De Boelelaan 1105, 1081 HV Amsterdam, The Netherlands; Research Institute for the heritage and history of the Cultural Landscape and Urban Environment (CLUE), Faculty of Arts, VU University Amsterdam, De Boelelaan 1105, 1081 HV Amsterdam, The Netherlands

**Keywords:** Niche construction theory, Hominins, Environmental management strategy, Social and environmental history, Central Netherlands

## Abstract

This paper shows what a niche construction theory (NCT) approach can contribute to the long-term social and environmental history of an area when applied to both sedentary and non-sedentary communities. To understand how communities create and respond to environmental change, hominin presence of the central Netherlands within the last 220,000 years is used as a case study. For this case study we studied the interrelationship between hominins, water and landscape gradients for four periods of interest within this long-term hominin presence. During each of these periods the study area had a specific environmental setting and (possible) traces of hominin presence. These periods cover the (1) Middle to Late Saalian (~220–170 ka), (2) Late Glacial (~14.7–11.7 ka, (3) Mid-Holocene (6000–5400 BP) and (4) Late Holocene (1200–8 BP). This review shows that traces of niche construction behaviour related to water and landscape gradients in the central Netherlands can be shown for both sedentary and non-sedentary communities. Furthermore, in this review it is shown that the transition from inceptive to counteractive change in ecosystem management style in the central Netherlands took place between the Mid- and Late Holocene periods.

## Introduction

**Fig. 1:**
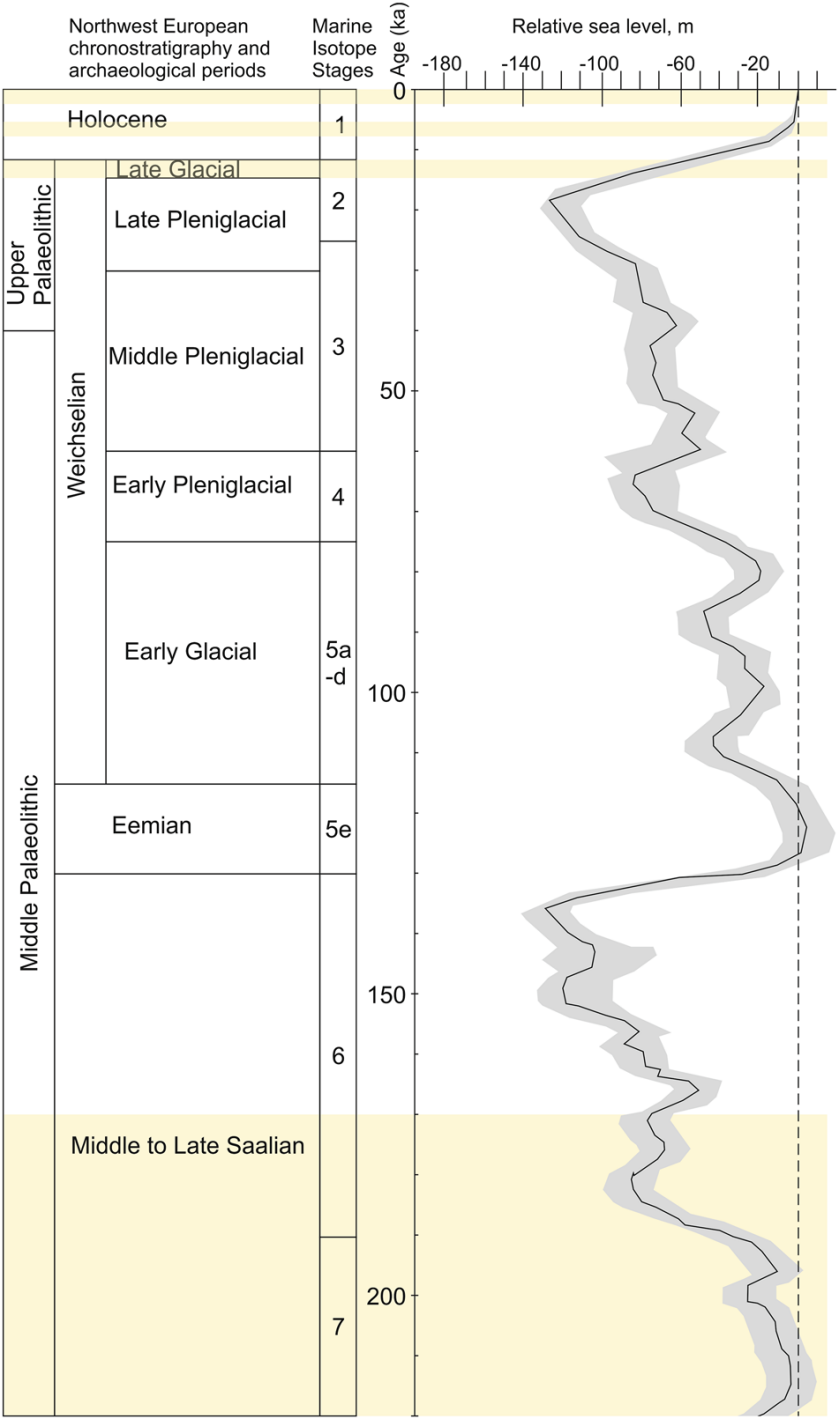
Northwest European chronostratigraphy, archaeological periods, climatic history and mondial relative sea level (RSL) record of the last 220,000 years. Chronostratigraphy according to Vandenberghe (1985) and Van Huissteden and Kasse (2001). Marine Isotope Record after Bassinot et al. (1994). RSL record after Waelbroeck et al. (2002). Error envelope of RSL from original source. *Yellow bars* indicate periods discussed in this paper

**Fig. 2:**
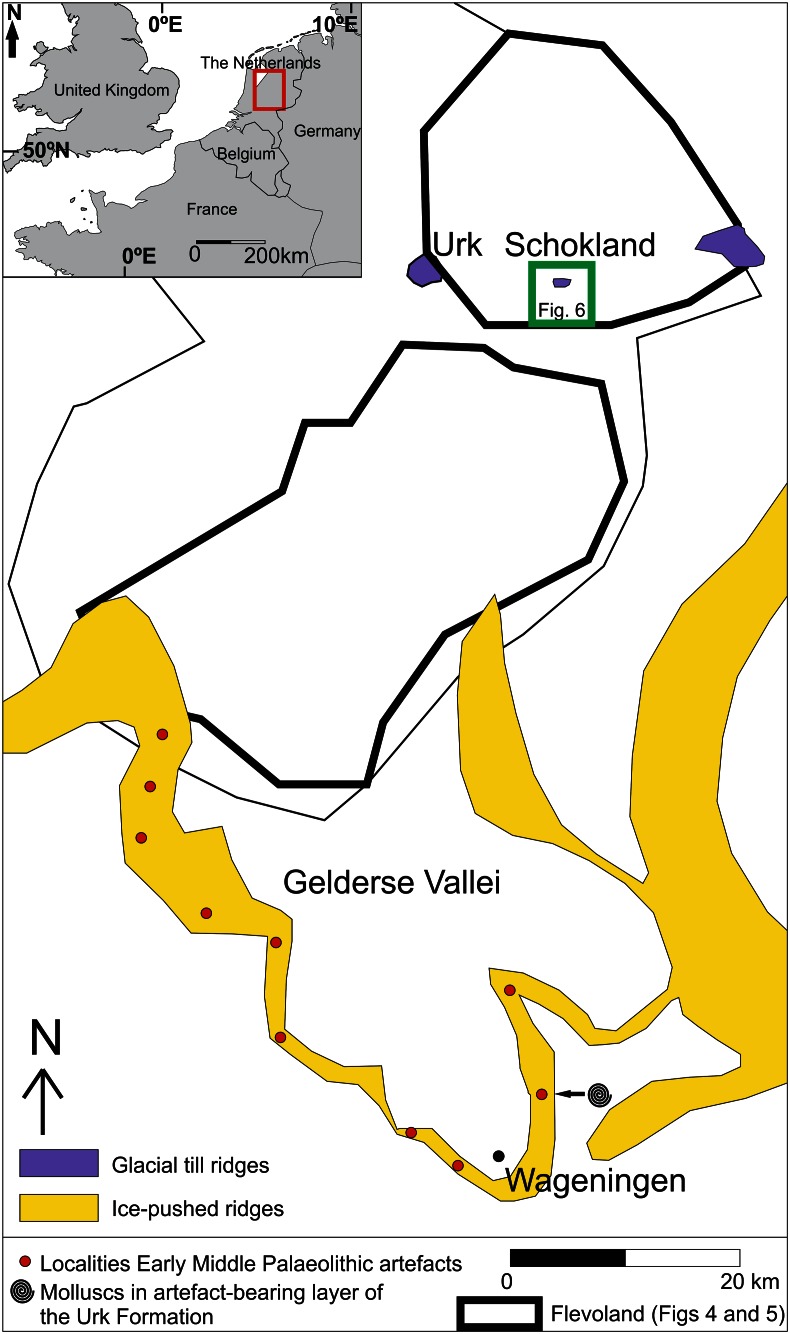
Overview of ice-pushed ridges (after Busschers et al. 2008; Van den Berg and Beets 1987), glacial till ridges (after Brouwer 1950; Busschers et al. 2008) and localities of Early Middle Palaeolithic (EMP) artefacts in the central Netherlands. Distribution of EMP artefacts after Stapert (1987) and Van Balen and Busschers (2010). *Inset* shows the location of the study area within the Netherlands

In the Netherlands there is a long-lasting tradition of living and struggling with the threat of water. The inhabitants of the coastal areas for example, faced a rapid rise in sea level during the Holocene (~last 11,700 years) (see Behre 2007; Jelgersma 1961, 1979; Van de Plassche 1982 for Holocene sea level curves of the Netherlands). This sea level rise was the result of melting of the glacial ice sheets since the end of the Last Glacial Maximum (~18,000 years ago) (Fig. 1) (see Simpson et al. 2009). One of the coastal areas in the Netherlands where water directly influenced the daily lives of its inhabitants was the Province of Flevoland (central Netherlands) (Fig. 2). During the last 1200 years for example, the area transformed from a peatland to an inlet of the North Sea (see Van den Biggelaar et al. 2014), until its reclamation from the sea between AD 1939 and 1968. This transformation is related to relative sea level rise, which was partly caused by surface lowering due to peat reclamation. As a response to the relative sea level rise, embankments were constructed at the former island Schokland in the northern part of Flevoland since ~750 BP (750 years before present (with present defined as AD 1950)) (Fig. 2) to protect its inhabitants against the increasing influence of the North Sea (Hogestijn 1992; Van der Heide and Wiggers 1954). This example of human-induced landscape transformation from Flevoland shows that organisms have the capacity to change their environment (for other examples see Hansell 1984; Jones et al. 1994, 1997, Laland et al. 1999, 1996; Lewontin 1983; Odling-Smee 1988; Odling-Smee et al. 1996), a process referred to as ‘niche construction’(Laland et al. 1999, 1996; Odling-Smee 1988; Odling-Smee et al. 1996). Apart from shaping their environment an organism-induced modification can also change other agents’ selective environment (Laland et al. 1999, 1996; Laland and Sterelny 2006; Odling-Smee et al. 2003).

Studies that deal with niche construction theory (NCT) applied to humans have focused predominantly on agricultural-based communities (e.g. Bleed 2006; Briggs et al. 2006; Redman 1999; Smith 2007a, 2011). However, recent archaeological research indicated that hunter-gatherers also affected their surroundings, although on a small scale (i.e. forager day-range) (e.g. Bliege Bird et al. 2008; Bos and Urz 2003; Bos et al. 2013; Pyne 1998).

In the context of the long-term changing interrelationship between hominins and their environment, we should go beyond modern *Homo sapiens* niche construction. Although evidence of pre-modern *Homo sapiens* niche construction is difficult to determine, it can be argued that they must also have had an impact on their environment (for examples of Neanderthal niche construction behaviours see Riel-Salvatore 2010). This impact is most likely similar to that of human hunter-gatherers (small-scale). Important features in the local habitat of hominins are water and landscape gradients (Kluiving, this issue). Landscape gradients provide for a wide variety of natural resources and water is the basis for life (see Rockström et al. 2014 for the importance of water for people). According to NCT, changes in the local habitat will affect hominin culture and consequently, a change in hominin life and economy can also modify the environment. Therefore, knowledge on the changing interrelationship between hominins, landscape gradients and water over time is fundamental for the understanding of the landscape and habitation history of an area.

However, studies that combine hominin-environment interaction of hunter-gatherer, agricultural and industrial communities do not yet exist.

Therefore, the aim of this review is to show how NCT can be applied on both sedentary and non-sedentary communities to increase our understanding of the long-term social and environmental history of an area.

**Fig. 3:**
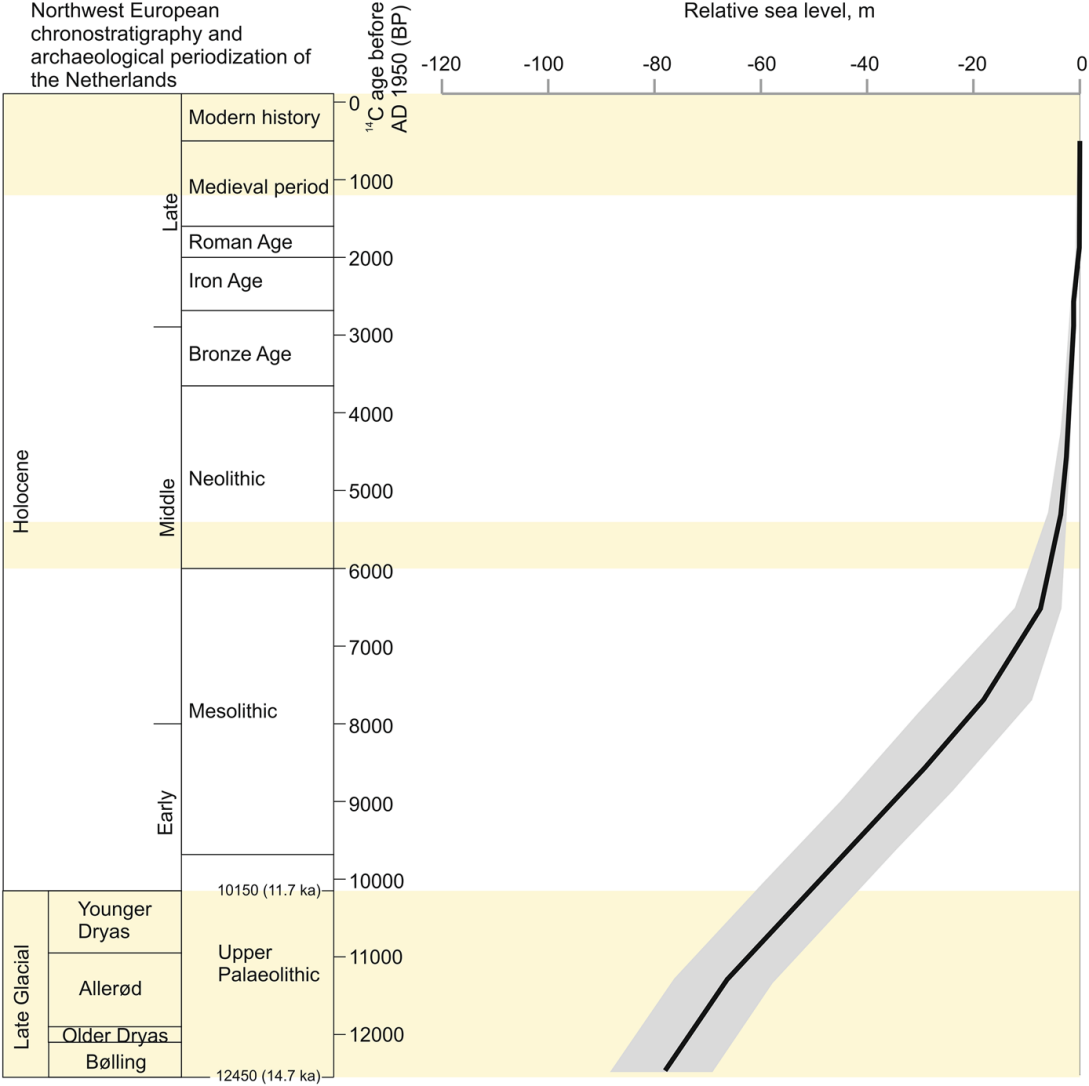
Northwest European chronostratigraphy, archaeological periodization of the Netherlands and mondial relative sea level (RSL) record of the Late Glacial and Holocene. Late Glacial chronostratigraphy according to Hoek (2001) and Lowe et al. (2008). Archaeological periodization after Louwe Kooijmans et al. (2005). RSL record after Waelbroek et al. (2002). Error envelope of RSL from original source. *Yellow bars* indicate periods discussed in this paper

The long-term hominin presence of the central Netherlands (Fig. 2) provide a case study to better understand the social and environmental history of the area. For this case study four periods of investigation are selected within the last 220,000 years which contain important transformations in the landscape and have (possible) traces of hominin presence (Van den Biggelaar, in prep.). These periods are (1) Middle to Late Saalian period (~220–170 ka) (ka = thousands of calibrated years before AD 1950), (2) Late Glacial period (~14.7–11.7 ka/ ~ 12,500–10,000 BP) (Late Glacial ages after Hoek 2001 with modifications from Lowe et al. 2008), (3) Mid-Holocene period (6000–5400 BP) and (4) Late Holocene period (1200–8 BP) (Figs. 1, 3). For each of these four periods an overview is given of the interrelationship between hominins, water and landscape gradients of the study area fixed at that time. The time frames between these time periods are discussed briefly. To study this long-term interaction a human (here, hominin) niche construction (HNC) approach was used.

### Palaeogeographical context and habitation history

#### Period 1: Middle to Late Saalian (~220–170 ka)

Prior to the maximum southward extension of the Fennoscandian ice sheet, corresponding to Marine Isotope Stage 6 (MIS 6, ~150 ka) (cf. Bassinot et al. 1994), the central Netherlands was part of a large delta. Before the land ice reached this landscape the area was inhabited by hunter-gathers. The southward ice advance (cf. Van den Berg and Beets 1987) formed ice-pushed ridges and glacial till ridges in the central Netherlands (Fig. 2) (e.g. Brouwer 1950; De Waard 1949; Jelgersma and Breeuwer 1975; Maarleveld 1983; Ruegg 1983; Ter Wee 1962, 1983; Van den Berg and Beets 1987; Wiggers 1955).

The Early Middle Palaeolithic (EMP) flint artefacts left by the early inhabitants of the central Netherlands occur in ice-pushed ridges surrounding the Gelderse Vallei area (Fig. 2). These ridges contain pushed alluvial deposits from the rivers Rhine and Meuse. Gravel and heavy mineral analyses indicated that the Rhine dominated the combined Rhine-Meuse fluvial system in the area (Busschers et al. 2008). However, the Meuse transported flint suitable for the production of tools towards the central Netherlands (Van Balen et al. 2007).

Early Middle Palaeolithic artefacts could possibly be present in the province of Flevoland (see Van den Biggelaar et al. in review), initiating the beginning of the biography of Flevoland (Van den Biggelaar in prep.). These artefacts possibly date between ~220 and ~170 ka (MIS 7—early MIS 6) (see Van den Biggelaar et al., in review). During MIS 7—early MIS 6 both warm and cool climatic phases occurred (Lisiecki and Raymo 2005), suggesting that the early hominins and their archaeological remains may be attributed to a variety of climatological and environmental settings.

In between periods 1 and 2 from the Late Saalian to the Late Pleniglacial (early MIS 6—late MIS 2, 170–14.7 ka), the climate is characterized by alternating cold and short-term temperate phases (e.g. Lisiecki and Raymo 2005; Van Huissteden and Kasse 2001; Zagwijn 1961). The first traces of hominin presence in the central Netherlands after the EMP date to the Weichselian (MIS 5d–2, 115–11.7 ka) (e.g. Johansen et al. 2007; Koopman et al. 2013; Schlüter 2003; Stapert 1980, 1991b, 1993; Van Uum and Wouters 1991). Except during maximum southward ice advance (~150 ka) and during the warm Interglacial Eemian (130–115 ka), the Rhine fluvial system dominated Flevoland until ~40 ka (Busschers et al. 2007; Peeters et al. 2014). After the Rhine abandoned the area, the sedimentary environment of the study region was dominated by aeolian coversand deposits (Spek et al. 1997, 2001a, b; Wiggers 1955).

#### Period 2: Late Glacial (~14.7–11.7 ka)

At the onset of the Late Glacial (LG) (~14.7 ka) (Fig. 3), Late Palaeolithic hunter—fisher—gatherer groups (Magdalenian, Creswellian and Hamburgian) inhabited the margins of upland areas in NW Europe (Terberger et al. 2009). After the arid and cool start of the LG, the Allerød interstadial (13.9–12.9 ka) is a relatively warm period during which woodlands formed and soils developed (e.g. Hoek 1997; Walker et al. 1994). During the Allerød (13.9–12.9 ka), NW Europe was inhabited by Federmesser hunter—fisher—gatherers. Federmesser sites are primarily concentrated at palaeolakes, fens and river terraces (e.g. Crombé et al. 2013, 2011; De Bie and Caspar 2000; Deeben 1988).

During the subsequent cool and arid Younger Dryas (YD) (12.9–11.7 ka) Ahrensburgian hunter—fisher –gatherer groups appear to concentrate predominantly at margins of upland areas (ridges and terrace edges) in close proximity to freshwater sources (Vermeersch 2011).

**Fig. 4:**
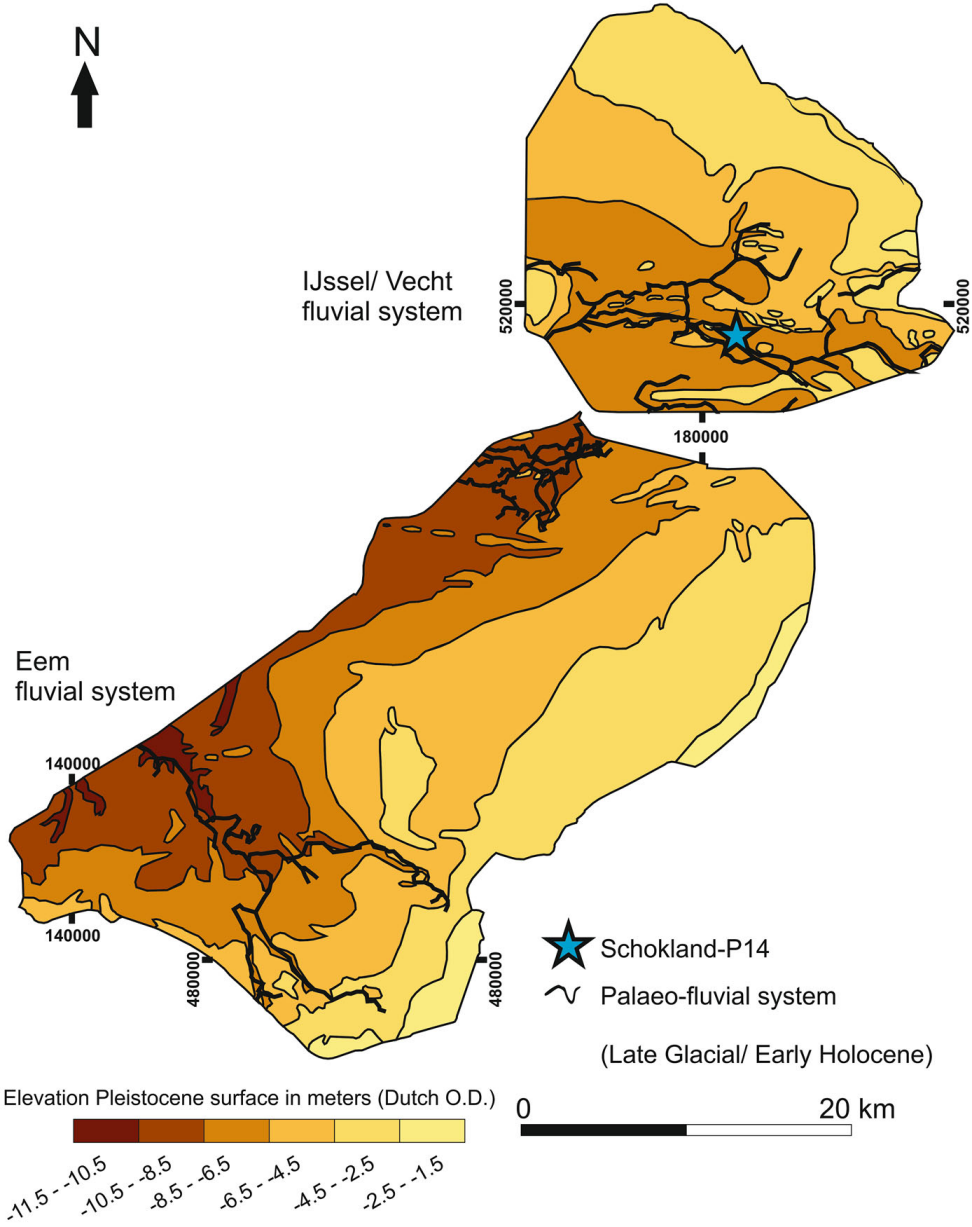
Elevation Pleistocene surface of Flevoland in meters Dutch O.D. (adapted from Peeters 2007) and location of palaeofluvial systems (compiled from Dresscher and Raemaekers 2010; Ente et al. 1986; Menke et al. 1998; Peeters 2007; Van den Biggelaar et al. 2015; Wiggers 1955). Location of the possible Late Glacial archaeological site Schokland-P14 after Ten Anscher 2012. For location of Flevoland in the Netherlands see Fig. 2

**Fig. 5:**
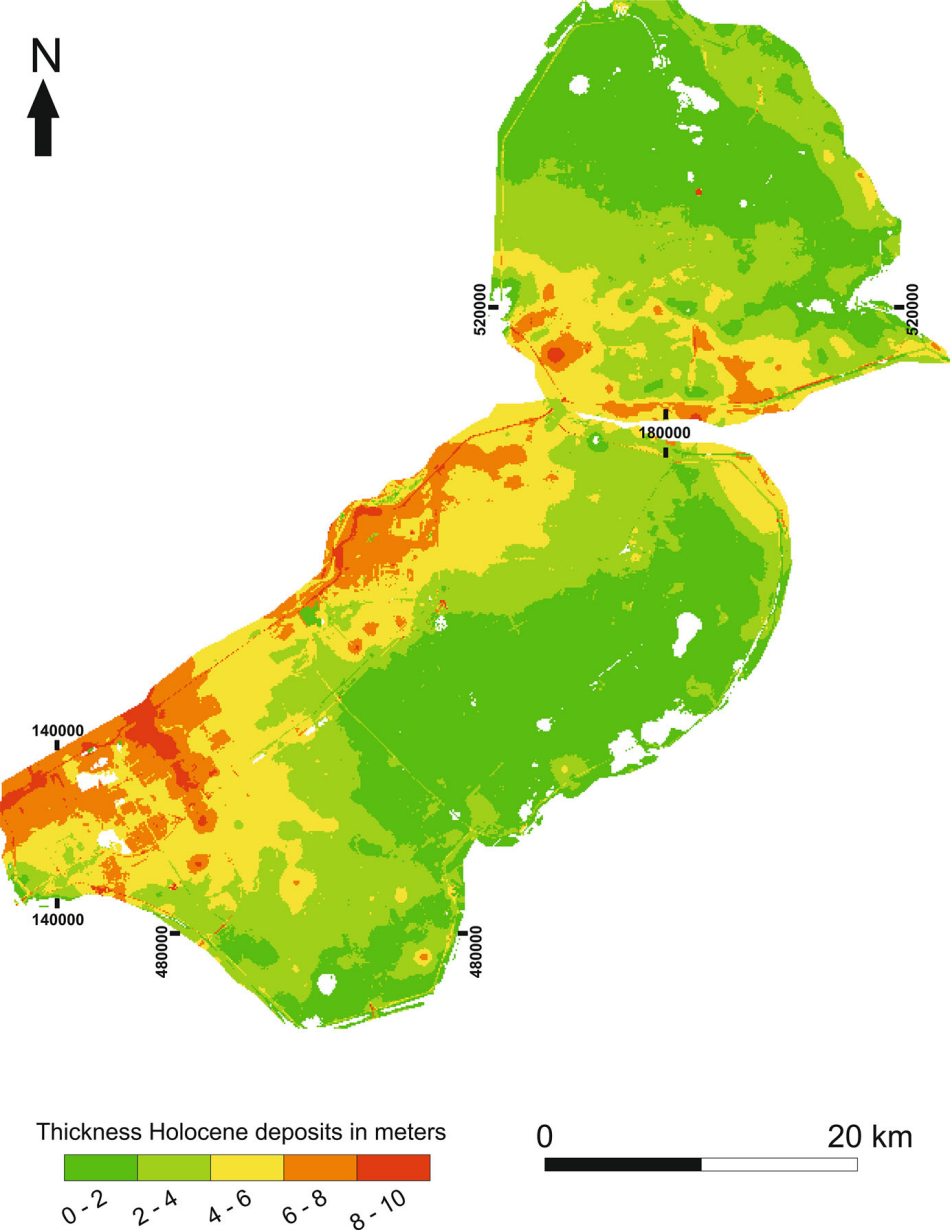
Thickness of Holocene deposits in Flevoland. Data from the Digital Geological Model (DGM) of the TNO Geological Survey of the Netherlands database (www.dinoloket.nl). For location of Flevoland in the Netherlands see Fig. 2

In the study region, the Eem and IJssel/Vecht fluvial systems were present during the LG. During the YD, dunes formed along the banks of the fluvial systems (Menke et al. 1998; Wiggers 1955). During this period the landscape is characterized by deeply incised gullies and high elevated dunes and ridges (e.g. Menke et al. 1998; Peeters 2007; Wiggers 1955; Van den Biggelaar et al. accepted). The gullies, dunes and ridges formed the undulating topography of the Pleistocene surface in the region (Peeters 2007; Van der Heide and Wiggers 1954; Wiggers 1955). This surface is sloping down in western direction and its elevation ranges from −11.5 to −1.5 m Dutch Ordnance Datum (D.O.) (Fig. 4). This undulating topography, together with the presence of freshwater sources indicates the regions’ potential for the availability of LG archaeological remains. This potential is further supported by the presence of LG archaeological remains surrounding the Flevoland region and the presence of intact Allerød soils and peat deposits in the study area (e.g. De Moor et al. 2013a, b; Van Smeerdijk 2002; Wiggers 1955). However, apart from a single possible Late Glacial archaeological site in Flevoland (Schokland—P14; see Ten Anscher 2012) (Fig. 4), there are no archaeological remains of this period in the region. The lack of archaeological remains dating to the LG is possibly a research bias. The study area is covered by 1 up to 10 meters of Holocene deposits (Fig. 5), making it almost impossible to retrieve LG archaeological remains.

The landscape processes in between period 2 and 3 are steered by climate amelioration at the onset of the Holocene (11.7 ka/ ~ 10,000 years BP), which resulted in extended soil formation in the top Pleistocene deposits. In the study region, the earliest Mesolithic traces that were left on these deposits date to around 9300 BP (Hamburg et al. 2012). Due to groundwater table rise, induced by postglacial sea-level rise, the Pleistocene surface was covered by peat that stopped Early Holocene soil formation (Havinga 1962; Hogestijn and Peeters 2001; Menke et al. 1998; Peeters 2007; Spek et al. 2001a, b; Wiggers 1955).

#### Period 3: Mid-Holocene (6000–5400 BP)

Continuing rise in the local groundwater level resulted in increasing tidal influence at the western part of the study area from ~6150 BP onwards (Ente 1971). Due to this tidal influence the western part of the area transformed from a peatland into a freshwater area with levees (Ente 1971, 1976; Ente et al. 1986; Menke et al. 1998; Van Zeist and Palfenier-Vegter 1981). However, some marine influence was also present as indicated by the presence of the clayey levees (Ente 1971; Schepers 2014b), coastal diatoms and some foraminifera (see Schepers 2014a for a literature overview and discussion on these palaeo-ecological indicators as a signal for incidental marine influence in the area). The combination of a dominant freshwater area with some marine influence can be described as a freshwater tidal area (cf. Schepers 2014b).

The Swifterbant culture that developed out of the Mesolithic hunter-gatherer groups (Deckers 1982; Louwe Kooijmans 1993; Whallon Jr and Price 1976), inhabited the study region from 6000 BP onwards (Louwe Kooijmans 1993; Peeters 2007; Raemaekers 1999, 2005; Van Gijn and Louwe Kooijmans 2005). Archaeological remains of the Swifterbant Culture in the study area are concentrated on Saalian glacial till ridges, Late Pleniglacial and Late Glacial coversand ridges and plateaus, Late Glacial source-bordering aeolian dunes and Mid-Holocene tidal levees (e.g. Hogestijn and Peeters 2001; Huisman et al. 2009; Peeters 2007; Raemaekers 1999, 2005; Van den Biggelaar et al. 2015).

The subsistence economy of the Swifterbant culture is characterised by a combination of hunting, gathering, fishing, domestication of animals and crop cultivation (extended broad spectrum economy) (Louwe Kooijmans 1993; Peeters 2007; Raemaekers 1999, 2005). The earliest traces of the domestication of animals in the area is dated to 5700 BP (Raemaekers 1999, 2005) and that of crop cultivation around 5400 BP (e.g. Huisman et al. 2009; Raemaekers 1999; Ten Anscher 2012). At that time, habitation in the area is concentrated on the higher elevated ground of the Eem and IJssel/Vecht fluvial systems (e.g. De Roever 2004; Hacquebord 1974, 1976; Raemaekers 2005; Van den Biggelaar et al. 2015).

Between periods 3 and 4 habitation continued to concentrate on the higher grounds until the Early Iron Age (2500 BP) (e.g. Gehasse 1995; Hogestijn 1991; Ten Anscher 2012; Ten Anscher and Gehasse 1993), due to continuous relative sea-level rise. A decrease in the rate of sea-level rise between 5500 and 3500 BP (Van der Spek and Beets 1992), resulted in the closure of tidal inlets along the Dutch western coast around 3200 BP (Berendsen 2008a; De Mulder and Bosch 1982; Vos et al. 2011). Subsequently, freshwater lakes within a peatland developed in Flevoland (Ente et al. 1986; Gotjé 1993; Pons and Wiggers 1960; Wiggers 1955). This poorly drained peat area had a low habitation potential. However, the discovery of modified wooden posts dating to the Early Roman period indicates that the area was visited around 1900 cal BP (Van Heeringen et al. 2014).

#### Period 4: medieval and modern period (1200–8 BP)

**Fig. 6:**
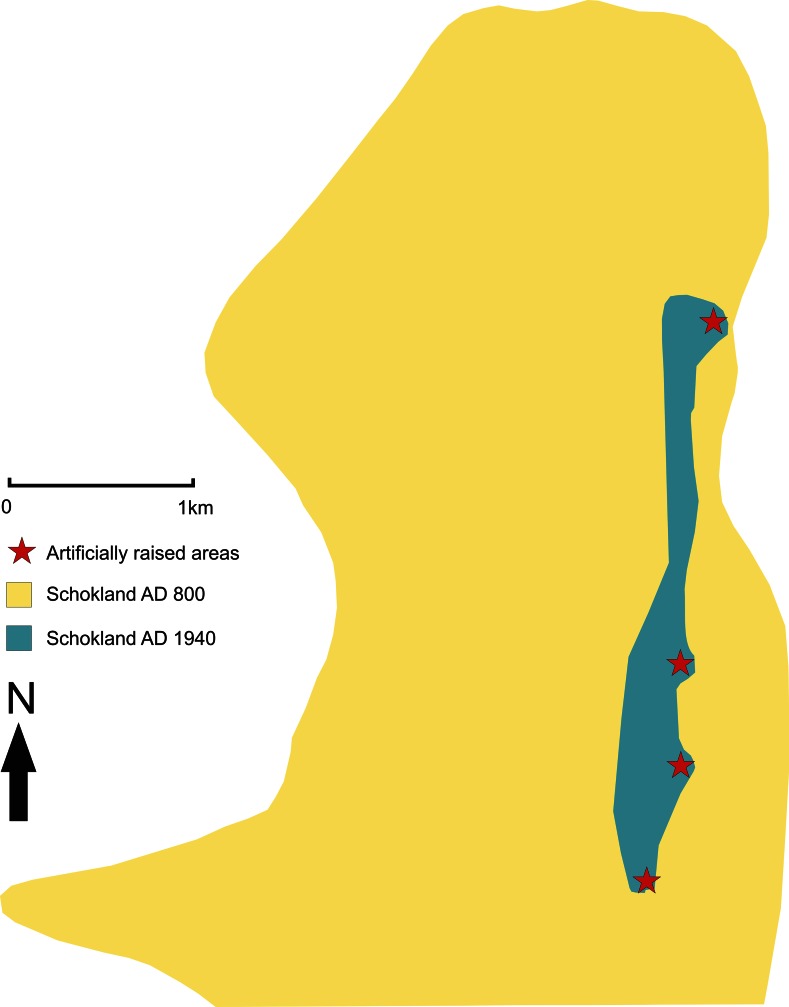
Extent of Schokland at AD 800 and AD 1940 and the location of the artificially raised areas (after Van der Heide and Wiggers 1954; adapted from Van den Biggelaar et al. 2014). For location of Schokland see Fig. 2

After 1200 BP, the peatland in Flevoland drained via the tidal inlet in the northwestern part of the Netherlands (Ente et al. 1986; Wiggers 1955). Due to this natural drainage, the habitation potential of the area increased (Van Balen 2008). Renewed habitation in Flevoland around 1150 BP (e.g. Hogestijn et al. 1994; Van der Heide and Wiggers 1954), led to the reclamation of the peatlands. Subsequent surface lowering due to oxidation and compaction of the peat (e.g. Hogestijn et al. 1994; Van der Heide and Wiggers 1954), caused increasing marine influence in the region. This resulted in erosion of the former island Schokland (northern part of Flevoland) by storm surges (Van den Biggelaar et al. 2014; Van den Biggelaar et al., in prep.). Consequently, unfavourable habitation conditions formed. To improve these conditions, the inhabitants of the former island constructed embankments since 750 BP (Hogestijn 1992; Van der Heide and Wiggers 1954) and moved to artifically raised areas around 500 BP (Fig. 6) (Van der Heide 1950; Van der Heide and Wiggers 1954). The marine environment dominated the region until the construction of the enclosure embankment in 18 BP (Wiggers 1955). The former island Schokland became a land-locked island after completion of the reclamation of the northern part of the study area at 8 BP (Wiggers 1955). The reclamation of the northern part of the study area involved creating fertile land. This area was inhabited over the past 70 years by an agricultural community. In AD 1995, Schokland became the 1st UNESCO World Heritage Site of the Netherlands and serves as an open-air museum since that time (www.schokland.nl; www.unesco.nl).

### Hominins, landscape gradients and water in Flevoland during the last 220,000 years

To understand how communities create and respond to environmental change, we focus on the role of water and landscape gradients in hominin communities within the last 220,000 years. The four periods discussed in this paper provide insight in hominin-water-landscape gradient interaction in both sedentary and non-sedentary communities in landforms as delta’s, river terraces, coastal estuaries and peat islands through time.

#### Early delta inhabitants

During the first period of interest (~220–170 ka), the central Netherlands was a large delta with intense fluvial dynamics and climate change over time. This delta had a high exploitation potential for hominins and animals due to the availability of freshwater. Furthermore, the fluvial systems dominating the area (Rhine: prior to 170 ka, combined Rhine/Meuse: ~ 170 ka; Busschers et al. 2008) most likely served as a corridor in the landscape. Also, the Meuse carried raw lithic material suitable for the production of artefacts (Stapert 1987, 1991a; Van Balen 2006; Van Balen et al. 2007). The Meuse transported this material from the southeastern Netherlands and adjoining areas towards the central Netherlands (Van Balen 2006; Van Balen et al. 2007). This fluvial landscape contained a plethora of natural resources. The faunal remains analysed in the artefact-bearing sediment in the study area indicate the presence of both temperate/warm and cold climate species. This co-occurrence of faunal remains of different climatic epochs is explained by the fluvial character of the deposits, hereby mixing sediments of different periods (Van Kolfschoten 1981, 1991). The cold climatic indicators are *Mammuthus primigenius*, *Coelodonta antiquitatis*, *Ovibos moschatus*, *Cervus (M.) giganteus*, *Rangifer tarandus* and *Bison priscus*. These species prefer an open steppe landscape. The composition of the temperate/warm climate fauna (*Elephas namadicus*, *Dicerorhinus kirchbergensis*, *Sus crofa*, *Cervus elaphus*, *Dicerorhinus bemitoechus*, *Equus* and *Hippopotamus amphibius*), indicate a wood/steppe biotope (Van Kolfschoten 1981, 1991). The wide variety of species during different climatic periods indicates the potential for exploitation in the area. Future research should indicate whether these species were exploited and if so, whether specific exploitation strategies were applied by EMP hominins (e.g. Neanderthals). One of those specific exploitation strategies by Neanderthals is selective hunting of prime-adult bovids (e.g. bison) (e.g. Gaudzinski 1995; Hoffecker et al. 1991) and cervids (e.g. reindeer) (e.g. Gaudzinski and Roebroeks 2000). Selective hunting of prime-aged prey can reduce the mean age at first reproduction in prey populations (see Stiner 1994). Earlier sexual maturity is one of the changes that is associated with the domestication of several mammal species, although the capacity for a species to be tamed is even more important (Belyaev 1979). Although selective hunting of certain mammal species cannot automatically be linked to the domestication process, it could have set the stage for the long complex process of domestication (Stiner 2002). The possible use of this specific exploitation strategy in the region indicates active modification of the sustainability of the natural resources by EMP hominins and can therefore be elaborated in a HNC approach.

Neanderthals not only applied a new flaking technology (Levallois technique), they were also the first hominins who distributed lithics across the landscape. This scattering of lithics for future use allowed them to adapt to a wide variety of ecosystems (e.g. Hovers and Kuhn 2006; Scott and Ashton 2011). This strategy allowed hominins to exploit less predictable and more widely spaced resources. In open environments for example, animal resources roam further than in forested environments. Therefore, hominins hunting these animals most likely adapted their strategy to be able to travel larger distances without the need of continuous access to raw lithic sources (Scott and Ashton 2011). Future research on the lithic distribution in the study area may indicate whether lithics were stockpiled to create a landscape scattered with lithics (sensu Webb 1993). This stockpiling indicates a change in the way Neanderthals engaged the landscape.

The fluvial system is not only an attractive area for hunting terrestrial animals, aquatic sources may also have been exploited. Both marine (e.g. *Haustator eryna*, *Scapharca diluvia* and *Venus multilamella*) and non-marine molluscs (e.g. *Valvata piscinalis*, *Bithynia tentaculata*, *Radix ovata* and *Corbicula fluminalis*) were found in the artefact-bearing sediment of the Urk Formation (combined Rhine-Meuse deposits) in the southern part of the study area near Wageningen (Fig. 2) (Meijer 1991). Although no evidence exists for the exploitation of aquatic sources in the region, in Spain marine resources (molluscs) have been systematically exploited by Neanderthals since ~150 ka (e.g. Cortés–Sánchez et al. 2011). Moreover, in France the earliest evidence of fish exploitation by Neanderthals date between 250,000 and 125,000 years ago (Hardy and Moncel 2011). The systematic exploitation of aquatic resources by Neanderthals since ~250 ka indicates that they were able to acquire fast-moving small prey, a trait previously seen as the domain of modern humans (Hardy and Moncel 2011). This suggests that Neanderthal resource exploitation may not be so different from that of *Homo sapiens*, indicating that just like *Homo sapiens*, Neanderthals were potentially seriously constructing hominin niches.

#### River terrace inhabitants

During the Late Glacial (~14.7–11.7 ka), elevated aeolian ridges and dunes are present within the Eem and IJssel/Vecht fluvial systems (see Menke et al. 1998; Peeters 2007; Wiggers 1955; Van den Biggelaar et al. accepted). Examples from the Netherlands and Northern Belgium show that such elevated areas in close proximity to a freshwater environment had a high habitation potential for Late Palaeolithic groups (e.g. Arts 1988; Bos et al. 2013; Crombé et al. 2013, 2011; De Bie and Vermeersch 1998; Deeben 1988; Derese et al. 2012; Vermeersch 2011). The combination of higher grounds and adjacent lakes and fluvial systems provided a high biodiversity. Also, the fluvial systems could be used as a corridor in the landscape. Furthermore, lithic sources were available in close proximity to the fluvial systems. The glacial till deposits at Urk and Schokland (northern part of Flevoland) and the ice-pushed ridges surrounding the Gelderse Vallei area (see Fig. 2) contain rocks (e.g. flint, granite and quartzitic sandstone) of useable size and composition for the production of tools (Devriendt 2014; Stapert 1981). The glacial till deposits of Urk and Schokland also contain amber that could be used for the production of tools (Van Spronsen 1977; Waterbolk and Waterbolk 1991). Although for the study area very few LG archaeological remains are known, the high potential of preserved LG archaeological remains (see Peeters 2007; Van den Biggelaar et al. accepted), indicates the regions’ high potential for future research on niche construction strategies. Examples of such strategies that have been documented for the LG at different parts of the world are: (1) systematic burning of fire for ecosystem engineering (e.g. Smith 2007b) and (2) domestication of plants and animals (e.g. Bleed and Matsui 2010; Yen 1989). The use of these strategies indicate an active modification of the exploitation potential of the landscape by its inhabitants and can therefore be applied in a niche construction approach.

#### Coastal area inhabitants

The gradual transformation of the study area into a wetland area with dry ridges and dunes during the Mid-Holocene (6000–5400 BP) (Ente 1971, 1976; Ente et al. 1986; Hacquebord 1976; Menke et al. 1998; Peeters 2007; Van den Biggelaar et al. 2015), resulted into a gradient-rich landscape with a variety of ecotones (such as back swamps, river banks and dunes; see Schepers 2014a for a complete overview). The diversity of ecosystems made the area suitable for an extended broad spectrum economy (hunting/gathering/fishing, domestication of animals and crop cultivation). There is a vast amount of literature concerning the introduction of crop cultivation (e.g. Bender 1978; Binford 1968; Bogucki 1988; Chase 1989; Cohen 1977; Flannery 1968; Hayden 1990; Ingold 1980; Keeley 1995; Rowley-Conwy 1984, 1985; Smith 1972; Sørensen and Karg 2012; Van den Biggelaar et al. 2015; Wright 1977; Zvelebil and Dolukhanov 1991). Although these studies might offer an explanation for the initial adoption of crop cultivation, NCT can provide insight in the way the landscape was domesticated to prepare for crop cultivation (Bleed and Matsui 2010). As suggested by Van den Biggelaar et al. (2015), the spatial differentiation of soil properties within the study area appears to have influenced choices of humans to adopt crop cultivation. The initial adoption of crop cultivation in the area is limited to areas with a high natural soil fertility (e.g. loam-rich glacial till ridges and clayey levees) (Van den Biggelaar et al. 2015). The rise in sea level resulted in increasing influence of the North Sea in the study region. Consequently, the sea deposited nutrient-rich clay in the area, forming the clayey levees that were used by the inhabitants for crop cultivation. This suggests that natural processes applied on a heterogeneous substrate are influencing NCT.

The use of an extended broad spectrum economy indicates that although domesticates (plants and animals) were used, these domesticated resources were not successful enough to stop people from hunting, gathering and fishing (Bleed and Matsui 2010). Future research on domesticates in the study area can provide insight in the factors that determine successful domestication.

Other examples of environmental management strategies in the region that are linked to water or landscape gradients and that could possibly have been applied are: (1) raising of habitation surface at tidal levees with reed bundles (Van der Waals 1977) and (2) burning of vegetation along the banks of the fluvial systems to improve fishing possibilites (Schepers 2014b).

#### Peat island inhabitants

For the Medieval and Modern Period (1200–8 BP) we focus on Schokland, because it is one of the few areas in Flevoland that was inhabited for the main part of that period. During this period water and landscape gradients play a dominate role in the human-environment response in the area.

Due to the natural drainage of Schokland since 1200 BP, the peatland became dry and the area had a high occupation potential. However, subsequent reclamation of the area since around 900 BP and possibly as early as 1150 BP (see Hogestijn et al. 1994), led to surface downwarping. This downwarping caused increasing marine influence in the area, opposite to what was anticipated with the reclamation of the area. Due to this marine influence the habitable area of Schokland and surroundings decreased over time (Fig. 6). Apart from modifications in the environment, the increasing marine influence also affected the subsistence economy of the inhabitants of Schokland. While crop cultivation and cattle were the main forms of subsistence until the 15th century (Geurts 1991; Van der Heide and Wiggers 1954), fishing became the main source of income until 91 BP when the former island was evacuated (Geurts 1991).

## Discussion

The most tangible traces of niche construction behaviour related to water and landscape gradients in the central Netherlands can be shown for the Mid-Holocene and Medieval and Modern Period. However, also for the Middle to Late Saalian and Late Glacial periods there is a wide variety of potential traces for environmental management strategies. While climate change is traditionally seen as the driving factor for the development of such strategies (e.g. Burroughs 2005; Richerson et al. 2001), NCT provides an important alternative (see Laland and O’Brien 2010).

During the Middle to Late Saalian, Early Middle Palaeolithic populations may have subjected the delta landscape to specific strategies exploiting faunal and aquatic resources. Together with stockpiling of lithics, these strategies indicate that EMP hominins were possibly seriously constructing hominin niches. Whether the population was able to change entire ecosystems still has to be debated, but those changes can be considered very small in terms of scale and assumed low impact on vast natural reserves. Although small-scale, the use of these strategies (e.g. stockpiling of lithics) indicates that EMP hominins were actively modifying their environment.

A niche construction approach on Late Glacial environmental management strategies (e.g. ecosystem engineering by fire and the domestication of plants and animals), opens up new avenues to investigate the development of these strategies into the Holocene. This approach could for example be used to understand the origin of domestication by taking into account the combination of changes in hominin behaviour, biology and ecology (see Laland and O’Brien 2010).

The Mid-Holocene coastal inhabitants (here: Swifterbant culture) expanded the hunter—fisher—gather subsistence economy with crop cultivation and the domestication of animals. The role of humans in the creation of a suitable ecology for the domestication of plants and animals provides a novel perspective to understand successful domestication (Bleed and Matsui 2010). This role most likely differs per location, because successful domestication has been documented for the Mid-Holocene for different environments across the world (e.g. Bleed and Matsui 2010; Lentz 2000; Terrell et al. 2003; Wagner 2003; Yen 1989). Although the choices of people influence successful domestication, the study by Van den Biggelaar et al. (2015) suggests that these choices are influenced by their natural environment (here: substrates with a high natural soil fertility).

The history of the peat island population of Schokland is determined by a loop of Human Niche Construction mechanisms that fits within the Anthropocene concept. This concept is used to describe the period during which human modification of the global environment outcompeted natural processes (see Crutzen 2002; Crutzen and Stoermer 2000). The starting date of the Anthropocene is under debate, ranging from the early Holocene (e.g. Smith and Zeder 2013) to AD 1945 (e.g. Zalasiewicz et al. 2014). For the western Netherlands the transition to the Anthropocene is placed around 3000 BP, based on the transition from a reactive to a proactive water management strategy, i.e. the transition from inceptive to counteractive changes (cf. Kluiving, this issue). Around 3000 BP peat development in the western Netherlands was at its greatest lateral expansion (see Vos et al. 2011). The dome shaped peat masses measured ~30 km^2^ and their top was located at an elevation of 5 m above current sea level (Eggelsmann and Schuch 1980). The increasing human interference in the landscape since 3000 BP resulted in natural erosion (Kluiving et al. 2013). The exploitation and excavation of peat for energy purposes in the 2nd millennium led to dewatering, oxidation and eventually considerable surface lowering (Berendsen 2008b; Van der Molen 1982). Currently, the lowest surface elevations in the western Netherlands are 5 m below Dutch Ordnance Datum (see Digital Elevation Model of surface elevation of the Netherlands, www.ahn.com), indicating that within 3000 years the actual peat surface has been lowered by 10 metres. This surface lowering initiated and enlarged the effect of relative sea level rise. These culturally induced natural processes testify that the (large scale) natural system is fundamentally altered, which in its turn alters ecosystems (cf. Kluiving, this issue).

At Schokland, the reclamation of the peatland around 900 BP (or as early as 1150 BP) (see Hogestijn et al. 1994) led to an unintentional change in the ecosystem, causing surface lowering. As a response to this change, water management strategies were applied (e.g. construction of embankments and raised areas). These strategies dominated the social life of the inhabitants of Schokland until the evacuation at 91 BP. The social and environmental history of Schokland since 900 BP is dominated by the interaction between its inhabitants and the increasing marine influence. This history was set in motion due to the unintentional change in the environment that was caused by hominin influence. Therefore, the Schokland example fits well within the concept of NCT. The similarity between Schokland and the western Netherlands in terms of culturally induced natural processes indicates that the environmental and cultural history of Schokland is a small-scale example of nation-wide relative sea level rise.

A comparison between the four investigated periods in terms of inceptive or counteractive ecosystem management style contributes to the discussion on the onset of the Anthropocene (see Kluiving, this issue for an overview of this discussion). For the Middle to Late Saalian and Late Glacial periods, societies exploited and adapted ecosystems on a small scale (forager day-range). The stockpiling of lithics and small-scale exploitation of specific resources during the Middle to Late Saalian indicate inceptive changes in the environment. The Late Glacial processes of anthropogenic fire and initial domestication of plants and animals are also examples of inceptive changes. Although during the Mid-Holocene societies possibly raised habitation levels with reed bundles (cf. Van der Waals 1977) and exploited the substrate on a landscape scale by crop domestication (e.g. Van den Biggelaar et al. 2015), these are still considered inceptive changes in NCT terminology. During the Late Holocene, cultural impact induced an unintentional macro-scale landscape change (peat surface lowering), which resulted in an unforeseen change in the ecosystem (enhanced effect of storm surges on Schokland). This process is an example of counteractive change in the environment. In summary, the transition from inceptive to counteractive ecosystem management styles occurred in Flevoland between the Mid- and Late Holocene periods. This supports the investigation by Kluiving (this issue) who placed this transition in the Western Netherlands around 3000 BP. The results of this study also indicate that traces of niche construction behaviour can be recognized for anthropogenic effects on ecosystems as early as the Middle to Late Saalian period (cf. Palaeoanthropocene concept of Foley et al. 2013). These results indicate that NCT allows to describe changes in hominin niche cycles such as inceptive to counteractive changes or scale differentiation of hominin impact. For this study a 3-stage temporal differentiation in scale of ecosystem management styles can be observed: (1) small-scale impact on ecosystems (Middle to Late Saalian, Late Glacial and most likely extending into the Holocene), (2) landscape domestication of preferred substrates on a landscape scale (Mid-Holocene), followed by domestication of the landscape on a supra-regional scale and (3) landscape transformation by flooding processes caused by human induced surface lowering of 10 meters during the Late Holocene (most drastic landscape changes).

To improve the understanding of the relationship between hominins and their environment a multi-disciplinary HNC approach is needed in which geoarchaeology plays an important role (see Kluiving, this issue). Interaction between hominins, water and landscape gradients in the central Netherlands covering the last 220,000 years indicate a wide variety of (possible) environmental management strategies and adaptations to natural ecosystem services. A HNC approach for the investigated periods provide new ways to evaluate the (geo)archaeological data to better understand the social and environmental history of the study area. Furthermore, a HNC approach can provide parameters of timing and duration of hominin impact on their environment in order to test its influence on large-scale eco-system dynamics. This insight plays and will play a key role in the current and future discussion of the Anthropocene concept.

## Conclusions

In this review we have shown that traces of niche construction behaviour related to water and landscape gradients in the central Netherlands can be shown for both sedentary and non-sedentary communities. Traces of observed and potential hominin niche construction behaviour in the central Netherlands can be divided into three scales of ecosystem management styles. During the Middle to Late Saalian and Late Glacial periods, societies exploit and adapt ecosystems on a small scale (forager day-range). Examples of potential ecosystem management techniques are stockpiling of lithics, anthropogenic fire and initial domestication of plants and faunal species. The Mid-Holocene societies adapted their preferred location of land management at a landscape scale in response to relative sea level rise. During the Late Holocene, the most drastic landscape changes took places on a macro-scale. Culturally induced natural processes (peat surface lowering) resulted in relative sea level rise, followed by an unintentional enhanced effect of storm surges in the area. The transition from inceptive to counteractive change in ecosystem management style in the central Netherlands took place between the Mid- and Late Holocene periods. Regional integrated case studies of geoarchaeological research provide for spatial and temporal reconstructions of the social and environmental history of an area and thereby contribute to the HNC approach.
